# The mediating role of motivation in the relationship between emotional intelligence and life satisfaction: an empirical study on teachers

**DOI:** 10.3389/fpsyg.2026.1652338

**Published:** 2026-01-28

**Authors:** Bünyamin Han

**Affiliations:** Education Faculty, Dumlupinar University, Kütahya, Türkiye

**Keywords:** emotional intelligence, life satisfaction, motivation, structural equation modeling (SEM), teachers, Türkiye

## Abstract

This study investigates the intricate relationship between emotional intelligence (EI), life satisfaction (LS), and the mediating role of motivation (M) within the context of Turkish teachers. Building upon extensive research on the interplay among these constructs, it is explored how EI, as a predictor, influences LS among educators, and whether M serves as a significant mediator in this relationship. The research draws upon a sample of teachers in Türkiye and employs structural equation modeling (SEM) to analyze the complex dynamics among these variables. According to the research results, EI positively affects LS and M, and at the same time, M plays a mediating role in the relationship between EI and LS. The findings of this study contribute to the understanding of the psychological well-being of teachers and may inform interventions aimed at enhancing their life satisfaction and motivation in the challenging educational landscape of Türkiye.

## 1 Introduction

Motivation, as a fundamental psychological process that drives, directs, and sustains individuals’ behavior, is a critical concept in understanding human behavior across many areas, from learning to job performance. The existing literature (Ryan and Deci, 2020; Yang, 2021; Mardanov, 2021; Akosile and Ekemen, 2022; Niemiec, 2024; Chiang, 2025) highlights the significant influence of both intrinsic and extrinsic motivation on personal satisfaction, individual growth, and self-determination, positioning motivation as a key determinant of human behavior and effort. However, more comprehensive and in-depth research is needed to fully illuminate the role of motivation in teachers’ individual achievement, goal attainment, and personal development, and its broader implications.

Motivational dynamics are thought to be closely related to various psychological constructs, particularly teachers’ emotional intelligence and life satisfaction. Research shows that these structures interact with each other and play an important role in understanding the complexity of human psychology (Ain et al., 2021; Helmi, 2021; Rogowska and Meres, 2022; Vilca-Pareja et al., 2022; Feraco et al., 2023). For example, emotional intelligence has the potential to shape both intrinsic and extrinsic motivations of individuals, which contributes significantly to their life satisfaction (Schutte et al., 2001; Extremera et al., 2011). However, the mechanisms of this complex relationship between EI, LS and M are still not fully understood and have not been sufficiently investigated. In parallel with this need, a more comprehensive theoretical and empirical examination of the interactions between these constructs is required to address the limitations of existing research and to better understand how motivational dynamics relate to emotional intelligence and life satisfaction. Accordingly, the aim of this study is to investigate the complex relationships between emotional intelligence, life satisfaction, and motivation, and to clarify how these reciprocal relationships affect teachers’ individual success and overall well-being.

### 1.1 Theoretical background

#### 1.1.1 Motivation

Motivation has been extensively studied through various theoretical frameworks, each providing distinct perspectives on its underlying mechanisms and determinants. Self-Determination Theory (SDT) highlights the dual nature of motivation, distinguishing between intrinsic and extrinsic forms, and underscores the importance of autonomy, competence, and relatedness in fostering motivated behavior (Deci and Ryan, 1985; Ryan, 2023; Wang et al., 2024). Expectancy-Value Theory (EVT) posits that motivation arises from an individual’s expectation of success and the subjective value they assign to a task or goal, emphasizing the interplay between cognitive appraisals and task importance (Eccles and Wigfield, 2002; Feather, 2021). According to the EVT, individuals prefer behavioral options that maximize the product of the actual value (incentive) and the probability of its occurrence (expectation; Beckmann and Heckhausen, 2025). Meanwhile, Goal Setting Theory (GST) asserts that establishing specific and challenging goals can significantly enhance both motivation and performance, demonstrating the role of goal clarity and difficulty in driving achievement (Locke and Latham, 1990; Bates et al., 2023). The theory’s core principles have been influential in shaping how organizations set goals to improve employee motivation, engagement, and performance (Ab Rahman, 2025). Together, these theories provide a comprehensive understanding of the multifaceted nature of motivation and its critical role in human behavior.

Motivation is influenced by a wide range of factors including *biological factors*; neurotransmitters such as dopamine and serotonin play a role in regulating motivation (Berridge, 2007; Freed, 2022); *psychological factors;* cognitive processes, such as self-efficacy and self-regulation, impact motivation (Bandura, 1986; Özen and Karaca, 2021; Schunk, 2023); *social and cultural factors;* social support, cultural norms, and peer influences shape motivation (Ryan and Deci, 2000; Markus and Kitayama, 1991; Zheng et al., 2021; Skinner et al., 2022); *educational context;* teachers’ feedback, classroom environment, and instructional strategies can impact student motivation (Ryan and Deci, 2017; Fong, 2022; Lu et al., 2022; Mirzaei et al., 2025); *workplace environment*; factors like recognition, job design, and organizational culture influence employee motivation (Basalamah, 2021; Ashfaq, 2024).

Understanding motivation has practical implications in various domains, such as education, work, and personal development. Motivated individuals are more likely to achieve their goals, perform better academically, and experience greater job satisfaction (Deci et al., 1991; Armenta et al., 2022; Latham, 2023; Feraco et al., 2023). Motivation is also an important tool to increase the efforts of employees in achieving organizational goals (Han et al., 2022; Karataş et al., 2025). Additionally, interventions aimed at enhancing motivation, such as goal setting and providing autonomy, have been implemented in educational and workplace settings (Ryan and Deci, 2017; Bates et al., 2023; Ab Rahman, 2025).

#### 1.1.2 Emotional intelligence

Emotional intelligence has become a prominent topic of interest in psychology and related fields over the past few decades. This multidimensional construct encompasses the ability to perceive, understand, regulate, and utilize emotions effectively, both in oneself and in others (Salovey and Mayer, 1990; Nelson and Low, 2011; Bru-Luna et al., 2021). EI is rooted in the notion that emotions play a crucial role in human cognition and behavior. Mayer and Salovey (1997) proposed a model that conceptualizes EI as a four-branch ability model, consisting of perceiving emotions, facilitating thought through emotions, understanding emotions, and managing emotions (Sharma and Pandey, 2024). Emotional intelligence is essential for physical and mental health, as well as for coping with situations where emotions play a significant role (Supramanian et al., 2021). Goleman (1995) expanded on this framework and introduced the idea of emotional competencies, emphasizing their significance in personal and professional success. The assessment of emotional intelligence has spawned a variety of instruments and models. One widely used tool is the Emotional Intelligence Appraisal developed by Travis and Greaves (2009), which measures EI in four domains: self-awareness, self-management, social awareness, and relationship management. Other measures include the Mayer-Salovey-Caruso Emotional Intelligence Test (MSCEIT; Mayer et al., 2002) and the Trait Emotional Intelligence Questionnaire (TEIQue) by Petrides (2009). However, the debate about the validity and reliability of these measures continues (Brackett and Mayer, 2003).

EI has been linked to various aspects of life, including interpersonal relationships, leadership, and well-being (Supramanian et al., 2021). Research suggests that individuals with higher EI are better equipped to navigate social situations (Brackett et al., 2006b; Jose and Thomas, 2024), exhibit more effective leadership (Goleman, 1998; Gómez-Leal et al., 2022), and experience better mental health (Schutte et al., 2007). In the workplace, EI is associated with job performance (Joseph and Newman, 2010), and career success (O'Boyle et al., 2011; Bansal and Bhattacharya, 2025), making it a valuable asset for employees and employers alike. Despite its popularity, EI is not without controversy. Critics argue that the concept is vague and lacks a unified definition (Matthews et al., 2002). There are concerns about the overemphasis on self-report measures, which may be subject to social desirability bias (Brackett and Mayer, 2003). Additionally, the question of whether EI can be taught and improved remains a topic of debate (Mayer et al., 2000). Since EI is the capacity of an individual to recognize, understand, regulate and use their own and others’ emotions effectively (Salovey and Mayer, 1990; Qin et al., 2023; Xu and Choi, 2023), this structure is expected to have a significant impact on teachers’ subjective well-being and life satisfaction.

#### 1.1.3 Life satisfaction

Life satisfaction, often referred to as subjective well-being or happiness, is a fundamental concept within the field of psychology. It represents individuals’ cognitive evaluations of their own lives, encompassing their overall sense of contentment, fulfillment, and happiness (Diener et al., 1985; Pavot and Diener, 1993; Huebner et al., 2006; Khodabakhsh, 2022). The concept of “satisfaction” mentioned here means that the person reaches inner satisfaction, that is, their desires come true (Elçiçek et al., 2022). The study of LS has gained prominence in psychological research, as it offers valuable insights into the factors that contribute to human flourishing and overall quality of life (Veenhoven, 2012; Armenta et al., 2022). LS is a complex and multifaceted construct, often conceptualized as comprising cognitive and affective components (Diener et al., 1985; Feraco et al., 2023). The cognitive component involves individuals’ cognitive judgments about their lives, such as how satisfied they are with various life domains (e.g., family, work, health). The affective component encompasses individuals’ emotional experiences, reflecting their overall emotional well-being and mood (Pavot and Diener, 1993).

LS is shaped by a complex interplay of personal, social, contextual, and psychological factors, each contributing uniquely to an individual’s overall well-being. Personal determinants, such as personality traits (e.g., extraversion and neuroticism), physical and mental health, and cognitive appraisals, play a significant role in influencing life satisfaction (Diener et al., 2002; Steel et al., 2008; Malvaso and Kang, 2022). Social factors, including social support, interpersonal relationships, marriage, and processes of social comparison, further underscore the relational dimension of life satisfaction (Diener and Seligman, 2002; Lyubomirsky and Layous, 2013). Contextual elements such as economic and employment status, income, and broader societal and cultural influences also significantly impact individuals’ evaluations of their lives (Easterlin, 2003; Inglehart and Klingemann, 2000). Additionally, psychological characteristics like optimism, gratitude, and resilience have been identified as crucial contributors, enhancing life satisfaction through their positive effects on emotional and cognitive processes (Seligman et al., 2005; Wood et al., 2008). Cheung et al. (2022) found that job satisfaction and overall work environment satisfaction are interconnected; a better work environment can increase job satisfaction, and vice versa. Together, these factors provide a multifaceted understanding of the determinants of LS, emphasizing its dynamic and multidimensional nature.

High LS is associated with numerous positive outcomes, including better physical and mental health, greater productivity, stronger social relationships, and enhanced overall well-being (Diener, 2009; Diener and Chan, 2011; Lyubomirsky et al., 2005; Arias et al., 2022; Poveda-Brotons et al., 2024; Rogowska and Meres, 2022). Conversely, low LS has been linked to increased vulnerability to psychological disorders, reduced life expectancy, and decreased overall life quality (Chida and Steptoe, 2008; Cheung et al., 2022).

### 1.2 The present study and the hypotheses

Since emotional intelligence refers to an individual’s capacity to effectively perceive, evaluate, and manage emotions, research findings suggest that these skills are related to individuals’ intrinsic and extrinsic motivation (Conde-Pipó et al., 2021; Chang and Tsai, 2022; Arias et al., 2022; Nieto Carracedo et al., 2024; Antonopoulou, 2024). Furthermore, the study of Chang and Tsai (2022) examined the relationship between EI and M, and reported that students’ EI had a positive effect on their learning motivation. Research shows that EI supports goal-oriented efforts by increasing individuals’ emotional awareness and improving their ability to cope with stress (Schutte et al., 2001). In addition, EI can function as a factor that strengthens individuals’ social bonds, helps them build supportive relationships and strengthen relationships (Gkintoni et al., 2025) and increases their motivation by enhancing their perception of social support (Bar-On, 2006; Poveda-Brotons et al., 2024). Moreover, EI strengthens individuals’ self-efficacy perceptions and social skills which increase their intrinsic motivation and helps them manage extrinsic motivations more effectively (Petrides and Furnham, 2001; Dumitru et al., 2025). According to the research of Arias et al. (2022), EI also increases students’ academic motivation. These findings reveal that emotional intelligence can be a basic psychological resource that positively affects motivation. Therefore, the first hypothesis of the research is as follow:

*H*1: Emotional intelligence positively affects motivation.

Research shows that individuals with high EI are more resilient in the face of emotional difficulties and exhibit better psychological adjustment (Schutte et al., 2001). This contributes to their feeling more satisfied with life. EI helps individuals develop empathy and social skills, allowing them to establish strong social bonds and build supportive relationships resulting increase in life satisfaction (Extremera et al., 2011; Rogowska and Meres, 2022; Qin et al., 2023; Xu and Wang, 2023; Alibabaie, 2025). In addition to individuals’ high capacity to cope with stress, EI also improves general psychological health and increases LS (Bar-On, 2006). According to Rogowska and Meres (2022) students with high levels of EI are better able to cope with challenges like Covid-19 and have higher levels of LS. In addition, the positive effect of EI on LS is related to individuals’ ability to manage negative emotional experiences in their lives, as well as the sense of success they provide in achieving personal goals. It has been shown that EI enables individuals to have a more satisfying life experience by increasing their motivation (Petrides and Furnham, 2001; Rogowska and Meres, 2022). Meta-analysis results also reinforce that emotional intelligence influences life satisfaction with cross-cultural, cross-ages samples (Helmi, 2021). A recent study also showed that higher emotional intelligence was also associated with a better quality of life, which in turn increased students’ life satisfaction (Alibabaie, 2025). In this context, EI can play an important role in determining the level of individuals’ ability to cope with emotional difficulties and their satisfaction with life. Therefore, the second hypothesis of this study was determined as follows:

*H*2: Emotional intelligence positively affects life satisfaction.

It is known that motivation directs individuals to live more satisfying and meaningful lives. Theories such as Self-Determination Theory (SDT) argue that conditions that improve individuals’ intrinsic motivation have positive effects on self-regulation, personal satisfaction, and quality of life (Deci and Ryan, 1985). Research findings suggest that individuals with high levels of M also have varying degrees of high LS (Armenta et al., 2022; Feraco et al., 2023; del Mar Salinas-Jiménez et al., 2024). Intrinsic motivation leads individuals to be more satisfied with the activities and highlights the role motivation plays in increasing life satisfaction or reducing stress (Meyer et al., 2021). In addition, it is seen that motivation enables individuals to experience meaningful success in the process of setting and achieving goals (Bhatt and Buddhapriya, 2021). Goal Setting Theory (GST) provides a framework that specifies the most valid and practical ways of increasing employee motivation (Latham, 2023). GST emphasizes that specific and challenging goals allow individuals to achieve higher levels of success and thus increase their LS (Locke and Latham, 1990). According to Latham (2023) achieving goals provides individuals with a sense of success, which increases emotional well-being and leads to a greater sense of satisfaction with life. It is also known that motivation helps individuals cope with environmental challenges and adopt a positive attitude (Budzanowska-Drzewiecka and Tutko, 2021). Motivation enables individuals to act based on their own internal strengths, independent of external factors, and to overcome difficulties in their lives more effectively and to achieve greater life satisfaction in the process (Ryan and Deci, 2000; Armenta et al., 2022; Feraco et al., 2023). Therefore, since individuals with high levels of motivation are expected to experience greater life satisfaction, the third hypothesis of the study was developed as follows:

*H*3: Motivation positively affects life satisfaction.

Research shows that EI enhances individuals’ ability to understand, regulate, and effectively utilize emotions, thereby strengthening both intrinsic and extrinsic motivation processes (Conde-Pipó et al., 2021; Chang and Tsai, 2022; Arias et al., 2022; Nieto Carracedo et al., 2024; Antonopoulou, 2024). Individuals with higher EI are more adept at maintaining goal-oriented behaviors, coping with challenges, and sustaining positive emotional states; all of which contribute to increased M. Increased M has been shown to lead to greater engagement, perseverance, and satisfaction in academic and personal areas (Arias et al., 2022; Poveda-Brotons et al., 2024), thus increasing LS. The study of Rogowska and Meres (2022) found that emotional intelligence is a significant positive predictor of job satisfaction and life satisfaction, and job satisfaction is a strong positive predictor of life satisfaction. In that case, motivation is expected to function as a mediating mechanism through which EI positively influences LS; this mechanism directs emotional competencies toward meaningful, goal-oriented actions that enhance overall well-being. Therefore, the fourth hypothesis of the study was developed as follows:

*H*4: Motivation has a mediating role in the relationship between EI and LS.

**Figure 1:**
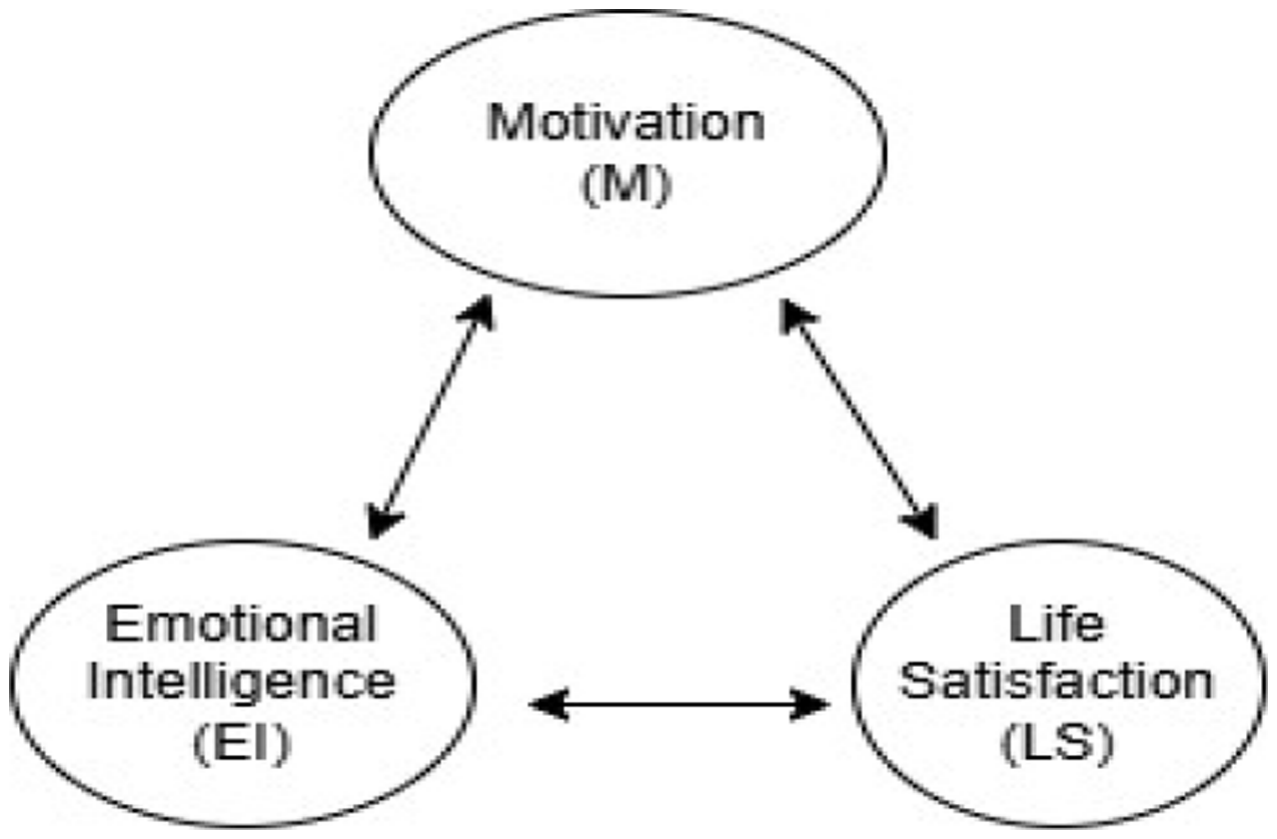
Hypotheses model of this research.

Figure 1 presents a hypothetical research model developed based on the literature examining EI, M, and LS. In the model, EI is proposed as a primary variable directly influencing both M and LS. M is positioned as a mediating variable, reflecting the assumption that individuals with EI are more capable of maintaining goal-oriented behaviors and commitment, thereby increasing their overall LS. Accordingly, the model includes both direct pathways (EI → LS; EI → M) and an indirect pathway (EI → M → LS), demonstrating the mediating role of M in the relationship between EI and LS. This conceptual framework forms the basis of the hypotheses tested in the study.

## 2 Method

This study is based on a cross-sectional research design aiming to examine the complex relationships among M, EI and LS among teachers in Türkiye. The research model provides a framework used to understand and analyze these relationships. In this section, further details will be provided to explain the methods used for data collection and analysis in order to support the study’s objectives and hypotheses.

### 2.1 The sample of the research

Participants were selected using stratified random sampling to ensure representation from diverse backgrounds and educational levels. The sample size is large enough to represent the population and consists of 239 teachers. Participants voluntarily participated in the research and their consent was obtained. Data were reported anonymized for analysis. Participants ranged in age from 25 to 55, with 130 male and 109 female teachers. Their seniority ranged from 2 to 20 years. In terms of education level, the majority held bachelor’s degrees (81%), while the number of postgraduate graduates was lower (19%). All participants worked in schools at the primary education level.

Since teacher appointments in Türkiye are made through central examinations, the socio-demographic distribution is heterogeneous. Therefore, even though the region where the data was collected was limited, the data possesses a diversity that allows for generalization across the country. These participants were selected to comprehensively understand the relationship between motivation (M), emotional intelligence (EI), and life satisfaction (LS) in the context of the teaching profession in Türkiye.

### 2.2 Data collection

The data of this research were collected by using 3 scales explained below.

#### 2.2.1 Emotional intelligence skills scale

This scale consists of 16 items and four dimensions developed by Wong and Law based on the theories of Salovey and Mayer (1990) on emotional intelligence. The first four items in the original scale are the dimension of being able to evaluate one’s own emotions, 5–8 items the dimension of evaluating the emotions of others, 9–12 items are exploiting emotions, 13–16 items are called the control of emotions dimension. The language and cultural adaptation of the scale into Turkish was conducted by Deniz et al. (2013). Language equivalence was supported by positive correlations between translated forms (English-Turkish) and reverse-translated forms (Turkish-English). Exploratory Factor Analysis for construct validity revealed that the scale is a four-factor measure consisting of 13 items. This four-factor solution was tested with Confirmatory Factor Analysis, and the results showed good fit with the data. The internal consistency score of TEIQue-SF is 0.81, and the test–retest reliability of the total score is 0.86. These results demonstrate that this scale is a valid and reliable instrument for use with Turkish university students (Deniz et al., 2013). Sample items from the scale include: “*It’s easy for me to talk about my feelings to other people*” (*Başkalarına duygularım hakkında konuşmak benim için kolaydır*) and “*I find it hard to control my feelings”* (*Duygularımı kontrol etmekte zorlanırım*).

#### 2.2.2 Motivation scale

The “Motivation at Work Scale,” originally developed by Gagné et al. (2010) and later adapted into Turkish both linguistically and culturally by Akbolat and Işık (2012) and Çivilidağ and Şekercioğlu (2017) was used to measure teacher motivation. This study used the Motivation at Work Scale (MAWS), developed by Gagné et al. (2010), which has been validated in both English and French versions. The original scale consists of four dimensions and 12 items. In the Turkish version, two dimensions, each with three items (intrinsic and extrinsic motivation), were validated. The Cronbach’s Alpha coefficient for the Turkish adaptation of the scale was found to be 0.904, indicating high internal consistency. Exploratory factor analysis (EFA) was performed to evaluate the validity of the scale. The suitability of the data for factor analysis was examined using the Kaiser-Meyer-Olkin (KMO) measure and the Bartlett Sphericity Test (BTS). The KMO value was found to be 0.904, indicating that the data were suitable for factor analysis. Furthermore, the BTS result was found to be significant, supporting the suitability of the factor model. Based on the explained variance, the number of factors to be retained was determined. The total variance explained by the scale was found to be 77.655%, indicating that the scale explains a significant portion of the variance in the data. Finally, Cronbach’s Alpha values were found to be above 0.70 for each dimension of the scale, indicating acceptable reliability across all dimensions (Akbolat and Işık, 2012). Sample items from the scale include: “*4. Bu iş amaçlarıma ulaşmamı sağlamaktadır” (4. This job allows me to attain my personal goals) and “10. Bu iş bana belirli bir yaşam standardı sağlamaktadır” (6. This job provides me with a certain standard of living).*

#### 2.2.3 Life satisfaction scale

This study employed the “Life Satisfaction Scale,” originally developed by Diener et al. (1985) and adapted into Turkish in terms of language and culture by Dağlı and Baysal (2016). The original scale was prepared in English and consists of five items with a single-factor structure. When adapting the scale, the Pearson product–moment correlation coefficient between Turkish and English versions was calculated and found to be 0.92. The scale’s Cronbach’s alpha internal consistency coefficient was 0.88, and the test–retest reliability was found to be 0.97. The factor analysis results revealed a single-factor structure, consisting of the same five items as in the original scale (Dağlı and Baysal, 2016). Examples of items on the scale include: *“3. Yaşamımdan memnunum” (3. I am satisfied with my life), “4. Şimdiye kadar yaşamdan istediğim önemli şeylere sahip oldum” (4. So far I have gotten the important things I want in life) ve “5. Tekrar dünyaya gelsem hayatımdaki hemen hemen hiçbir şeyi değiştirmezdim” (5. If I could live my life over, I would change almost nothing).*

### 2.3 Data analysis

In this study, the fit indices obtained from the Confirmatory Factor Analysis (CFA) for each scale used are presented in Table 1.

**Table 1:** Fit indices for scales.

Scales	x^2^	Sd	x^2^/sd	*p*	GFI	IFI	TLI	CFI	RMSEA
Emotional intelligence	131.86	59	2.23	0.00	0.91	0.95	0.95	0.95	0.07
Motivation	6.25	3	2.08	0.10	0.99	0.99	0.98	0.99	0.06
Life satisfaction	1.40	2	0.70	0.49	0.99	1.00	1.00	1.00	0.00

When Table 1 is examined, the x^2^/df ratio, which is between 1 and 3 for emotional intelligence, motivation, and life satisfaction variables, indicates the presence of a good fit. The RMSEA value, being above 0.05 for emotional intelligence and motivation variables, suggests an acceptable fit. For life satisfaction, the RMSEA value is less than 0.05, indicating a high level of fit. The GFI value is greater than 0.90 for all research variables, indicating a good fit (Kline, 2010). Furthermore, when examining IFI, TLI, and CFI values, being 0.95 or higher signifies a good fit (Byrne, 2013). The fit indices obtained from confirmatory factor analyses (CFA) performed for each research variable reveal that the factor structures of the scales used in the study show a good or acceptable level of fit with the research data.

The dataset underwent checks for skewness and kurtosis coefficients. It was observed that the tolerance value was greater than 0.2, and the VIF value was less than 10, indicating the absence of multicollinearity problems between dependent and independent variables. The data of the study were examined for validity and reliability. Confirmatory factor analysis demonstrated the fit between the utilized scales and the study’s data. Reliability analysis confirmed the reliability of each scale. Subsequently, in this research, measurement and structural models were constructed to provide stronger evidence for the relationships between variables.

## 3 Findings

**Figure 2:**
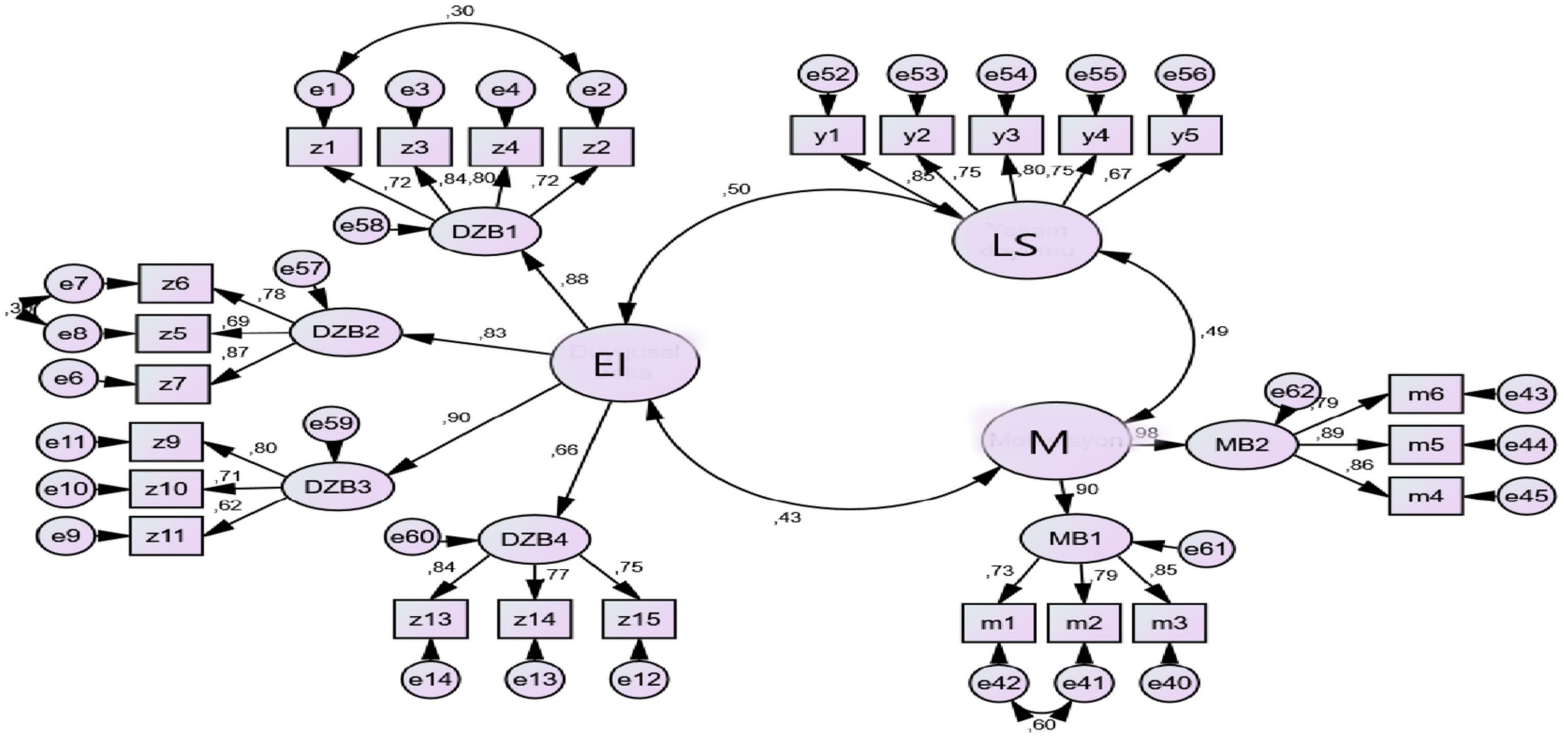
Measurement model.

The findings of this research are given below. In the measurement model, the relationships between latent variables are presented in Figure 2.

When examining the measurement model presented in Figure 2, error covariances have been added between the items z1 and z2, z5 and z6, and m1 and m2 due to the relatedness of errors of these items. The measurement model demonstrates that there is a significant relationship between the research variables. Upon reviewing the generated fit indices, it is observed that they are at a good and acceptable level (χ^2^ = 572.74, df = 240, χ^2^ / df = 2.38, *p* = 0.00, GFI = 0.85, IFI = 0.91, TLI = 0.90, CFI = 0.91, RMSEA = 0.07).

**Figure 3:**
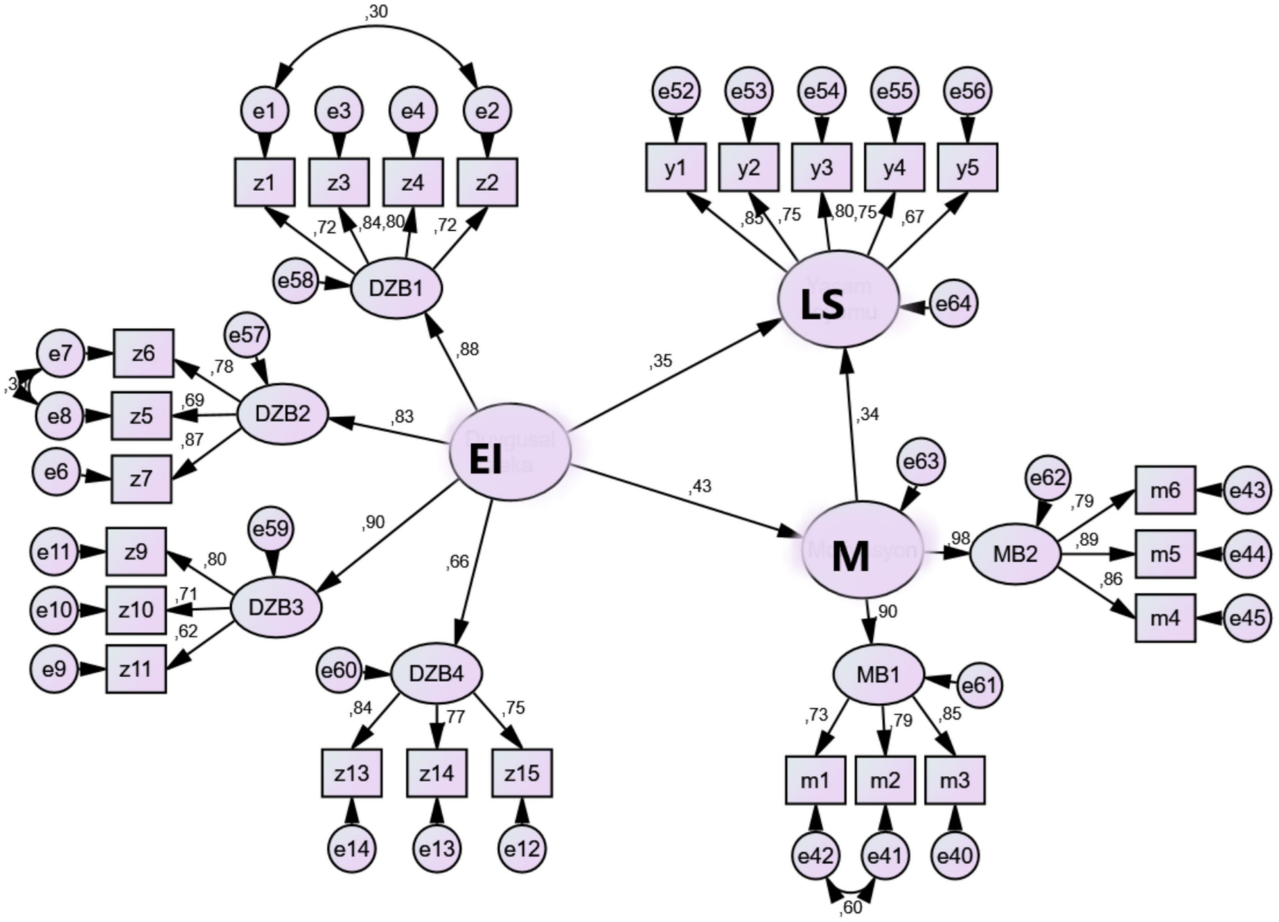
Structural equation model.

Covariances added between latent variables in the measurement model have been removed. Considering the hypotheses in this research, unidirectional paths have been added to the latent variables. It has been observed that the unidirectional arrows added in the proposed structural model have significant path coefficients. Therefore, no paths have been removed from the structural model (Figure 3).

When examining the fit indices generated in the structural model, it is observed that the data from the research is in an acceptable and good fit with the structural model (χ^2^ = 572.74, df = 240, χ^2^ / df = 2.38, *p* = 0.00, GFI = 0.85, IFI = 0.91, TLI = 0.90, CFI = 0.91, RMSEA = 0.07).

The emotional intelligence (EI) levels of teachers positively influence their motivation (M; ß = 0.43, *p* < 0.01) and life satisfaction (LS; ß = 0.35, *p* < 0.01). Motivation, in turn, positively affects life satisfaction (ß = 0.34, *p* < 0.01). With the addition of the motivation variable to the model, the effect of emotional intelligence on life satisfaction decreases significantly from 0.50 to 0.15. Thus, emotional intelligence positively affects life satisfaction both directly and indirectly through partial mediation by motivation (ß = 0.15, *p* < 0.01).

## 4 Results and discussion

The results of the study supported all four hypotheses, providing significant relationships between EI, M and LS.

This research analysis revealed a significant positive relationship between EI and M. Consistent with previous research, the findings of this study reaffirm that individuals with higher EI report greater levels of LS (Brackett et al., 2006a; Extremera and Fernández-Berrocal, 2005; Nelson and Low, 2011; Bru-Luna et al., 2021). Moreover, this study provides critical insights into the mechanisms underpinning the relationship between EI and LS. Motivation, recognized as a fundamental driver of human behavior emerges as a key mediating factor in this dynamic (Helmi, 2021; Rogowska and Meres, 2022; Alibabaie, 2025). The results highlight that EI not only directly enhances LS but also exerts its influence indirectly by fostering higher levels of motivation, which in turn positively impacts LS (Rogowska and Meres, 2022).

The findings of this study underscore the mediating role of motivation in the relationship between EI and LS. EI enhances an individual’s ability to manage emotions, improve interpersonal relationships, and effectively navigate social complexities (Brackett and Mayer, 2003; Qin et al., 2023; Xu and Wang, 2023). These competencies not only contribute to personal and social well-being but also serve as catalysts for motivation, driving individuals toward meaningful and fulfilling goals (Bandura, 2001). Motivation, whether intrinsic or extrinsic, plays a critical role in this dynamic (Deci and Ryan, 1985; Armenta et al., 2022; Feraco et al., 2023). According to Latham (2023) both forms of motivation are closely linked to LS, acting as pathways through which EI exerts its influence. By mediating the EI-LS relationship, motivation highlights the importance of understanding and fostering emotional and motivational processes in enhancing overall well-being. This interplay suggests that interventions aimed at boosting EI and cultivating both intrinsic and extrinsic motivation could significantly enhance LS.

The findings of this study emphasize the crucial role of M as a mediator in the relationship between EI and LS. Individuals with higher EI are better equipped to set and pursue intrinsic goals that align with their core values and personal interests (Mayer et al., 2008; Conde-Pipó et al., 2021; Chang and Tsai, 2022; Antonopoulou, 2024). Such individuals tend to exhibit greater self-regulation, resilience, and emotional self-awareness, all of which are essential for sustaining intrinsic motivation (den Van Broeck et al., 2016; Arias et al., 2022; Nieto Carracedo et al., 2024). This enhanced intrinsic motivation, driven by a strong sense of purpose, results in higher levels of LS (Huy and Shipilov, 2012). Moreover, EI also influences extrinsic motivation. The study of Chang and Tsai (2022) reported that students’ EI had a positive effect on their learning motivation. People with high EI possess the ability to navigate complex social environments, manage relationships effectively, and seek support when necessary, thereby increasing their potential to attain external rewards (Mikolajczak et al., 2007). Their proficiency in recognizing and regulating emotions in others enhances their capacity to garner resources, recognition, and social rewards, all of which contribute to their overall well-being and life satisfaction (Van Kleef et al., 2009; Rogowska and Meres, 2022). Meta-analysis results also reinforce that emotional intelligence influences LS (Helmi, 2021). This finding highlights the mediating role of M, both intrinsic and extrinsic, in the relationship between EI and LS. While prior research has established a direct link between EI and LS, this study provides a more nuanced understanding by uncovering the intricate pathways through which EI influences well-being. M, in its various forms, emerges as a pivotal factor bridging the gap between EI and LS. Considering these findings, it is evident that fostering EI can significantly enhance both intrinsic and extrinsic motivation, leading to improved LS.

## 5 Conclusion

This study has explored the intricate and multifaceted relationship between teachers’ M, EI and LS. This research has underscored the critical role of motivation as a mediating factor that mediates the relationship between EI and overall LS. This research reveals that individuals with higher EI possess a heightened capacity to regulate emotions, manage interpersonal relationships, and navigate social complexities (Brackett and Mayer, 2003). Teachers’ abilities in this area have been found to be closely linked to their motivation to achieve meaningful goals and aspirations. Motivation, whether intrinsic or extrinsic, acts as a conduit through which emotional intelligence channels its influence on LS. Intrinsic motivation, driven by a sense of personal values and a deep connection to one’s goals, appears to be fostered by EI (Mayer et al., 2008). Teachers who exhibit high emotional intelligence demonstrate greater resilience, emotional self-awareness, and self-regulation; these qualities strengthen their intrinsic motivation. As a result, they have been found to derive higher levels of life satisfaction from their professional endeavors. EI facilitates the attainment of external rewards and recognition through effective social navigation (Mikolajczak et al., 2007; Van Kleef et al., 2009). Individuals adept at recognizing and regulating others’ emotions can leverage these skills to garner external resources (den Van Broeck et al., 2016), which in turn contributes to high LS.

## 6 Practical implications

Understanding the interaction between motivation (M), emotional intelligence (EI), and life satisfaction (LS) offers valuable guidance for teachers’ professional development and well-being. Strengthening motivation can enhance key dimensions of EI such as emotional awareness, regulation, and resilience; which in turn contribute to higher levels of life satisfaction in teaching contexts. Educators and employers can use this knowledge to design environments that foster M, thereby enhancing EI and subsequently, LS. Schools and administrators can support this process by creating work environments that foster teachers’ intrinsic motivation, appreciate their efforts, and offer meaningful opportunities for professional autonomy and development. In particular, training programs aimed at developing emotional intelligence skills such as emotional regulation, empathy, and reflective practice can further enhance teachers’ motivation and overall well-being.

While we have delved into the complex interrelationships between M, EI and LS, there remain areas ripe for further exploration. Future studies should investigate the specific mechanisms by which M shapes EI and LS among teachers. Additionally, cross-cultural research could offer a deeper understanding of how best to support teachers globally by revealing whether these dynamics operate universally or vary across different educational and cultural contexts.

## 7 Limitations

While this study provides valuable insights into the relationship between EI and LS and the mediating role of M among Turkish teachers, several limitations should be considered. First, the sample is limited to teachers in Türkiye, which may affect the generalizability of the findings to teachers in other cultural or educational contexts. Although this study sample included teachers from diverse socio-cultural backgrounds, the cultural and socio-economic context specific to Türkiye may influence the dynamics of motivation, emotional intelligence, and life satisfaction in ways different from those in other countries. Thus, future research could consider cross-cultural comparisons to examine whether the results hold true across diverse populations. Second, the study relies on self-reported measures of M, EI and LS which can introduce biases such as social desirability or response styles. While structural equation modeling (SEM) offers robust statistical analysis, self-report data may not fully capture the complexity of these constructs, as it may be influenced by participants’ subjective perceptions rather than objective behaviors or outcomes. Using multiple methods, such as behavioral observations or third-party assessments, could provide a more comprehensive understanding of the variables under investigation. Third, this study explores the relationships among M, EI and LS at a single point in time, which limits its ability to draw causal conclusions. Longitudinal studies would provide more conclusive evidence regarding the directionality of these relationships and whether changes in EI or LS over time lead to improvements in M.

The study does not account for potential confounding variables that may influence the relationships these structures, such as individual differences in personality traits, work-related stressors. Future research could incorporate these factors to further clarify the mechanisms through which EI and LS influence M in teachers. While the current study contributes to understanding the teachers’ M, EI, and LS, these limitations highlight the need for further research to validate and expand upon the findings in diverse contexts and with more comprehensive methodological approaches.

## Data Availability

The data of this research will be made available on reasonable request.

## References

[ref1] Ab Rahman M. R. Z. B. (2025). Why goal setting theory is the most relevant in today’s workforce? Malays. J. Bus. Econ. Manag. 4, 41–48. doi: 10.56532/mjbem.v4i1.90

[ref2] Ain N. U. Munir M. Suneel I. (2021). Role of emotional intelligence and grit in life satisfaction. Heliyon 7, 1–7. doi: 10.1016/j.heliyon.2021.e06829 PMC8093457 33997383

[ref3] Akbolat M. Işık O. (2012). Sağlık çalışanlarının duygusal zekâ düzeylerinin motivasyonlarına etkisi. Dumlupınar Üniv. Sos. Bilim. Derg. 32, 109–124.

[ref4] Akosile A. L. Ekemen M. A. (2022). The impact of core self-evaluations on job satisfaction and turnover intention among higher education academic staff: mediating roles of intrinsic and extrinsic motivation. Behav. Sci. 12:236. doi: 10.3390/bs12070236, 35877307 PMC9311765

[ref5] Alibabaie N. (2025). A study on the relationship between quality of life, emotional intelligence and life satisfaction among students. Health Educ. Health Promot. 3, 3–13.

[ref6] Antonopoulou H. (2024). The value of emotional intelligence: self-awareness, self-regulation, motivation, and empathy as key components. Tech. Educ. Humanit. 8, 78–92. doi: 10.47577/teh.v8i.9719

[ref7] Arias J. Soto-Carballo J. G. Pino-Juste M. R. (2022). Emotional intelligence and academic motivation in primary school students. Psicol. Reflex. Crít. 35:14. doi: 10.1186/s41155-022-00216-0, 35622170 PMC9142720

[ref8] Armenta C. N. Fritz M. M. Walsh L. C. Lyubomirsky S. (2022). Satisfied yet striving: gratitude fosters life satisfaction and improvement motivation in youth. Emotion 22, 1004–1016. doi: 10.1037/emo0000896, 32915004

[ref9] Ashfaq R. (2024). Influence of workplace environment on employee motivation and employee productivity. Master thesis. Warsaw: Jagiellonian University Repository.

[ref10] Bandura A. (1986). Social foundations of thought and action. NJ: Englewood Cliffs.

[ref11] Bandura A. (2001). Social cognitive theory: an agentic perspective. Annu. Rev. Psychol. 52, 1–26. doi: 10.1146/annurev.psych.52.1.1, 11148297

[ref12] Bansal D. Bhattacharya N. (2025). “Navigating success: the intersection of emotional intelligence and career development” in Emotional intelligence in the digital era. eds. Dutta P. K. Gupta S. Kashyap S. Gehlot A. Karmakar R. Bhattachary P.. (New York: Auerbach Publications), 141–158.

[ref13] Bar-On R. (2006). The Bar-On model of emotional-social intelligence (ESI). Psicothema 18, 13–25. 17295953

[ref14] Basalamah S. A. (2021). The role of work motivation and work environment in improving job satisfaction. Golden Ratio Hum. Resour. Manag. 1, 94–103. doi: 10.52970/grhrm.v1i2.54

[ref15] Bates T. C. Enkhbat T. Gray E. Lee J. Zakharin M. (2023). How to get things done: tight linkage of conscientiousness with twelve mechanisms of goal setting theory. Pers. Individ. Differ. 214:112331. doi: 10.1016/j.paid.2023.112331

[ref16] Beckmann J. Heckhausen H. (2025). “Motivation as a function of expectancy and incentive” in Motivation and action. eds. Heckhausen J. Frontmatter H. H.. (Springer Nature Switzerland: Cham), 165–225.

[ref9001] Berridge K. C. (2007). The debate over dopamine’s role in reward: the case for incentive salience. Psychopharm 19, 391–431. doi: 10.1007/s00213-006-0578-x 17072591

[ref17] Bhatt P. Buddhapriya S. (2021). Intrinsic motivational potential and its effect on academic performance, life-satisfaction and procrastination: a study of MBA students. Int. J. Indian Cult. Bus. Manag. 22, 443–460. doi: 10.1504/IJICBM.2021.114995

[ref18] Brackett M. A. Mayer J. D. (2003). Convergent, discriminant, and incremental validity of competing measures of emotional intelligence. Personal. Soc. Psychol. Bull. 29, 1147–1158. doi: 10.1177/0146167203254596, 15189610

[ref19] Brackett M. A. Palomera R. Mojsa J. A. Reyes M. R. Salovey P. (2006a). Emotion-regulation ability, burnout, and job satisfaction among British secondary-school teachers. Psychol. Sch. 43, 319–337.

[ref20] Brackett M. A. Rivers S. E. Shiffman S. Lerner N. Salovey P. (2006b). Relating emotional abilities to social functioning: a comparison of self-report and performance measures of emotional intelligence. J. Pers. Soc. Psychol. 91, 780–795. doi: 10.1037/0022-3514.91.4.780, 17014299

[ref22] Bru-Luna L. M. Martí-Vilar M. Merino-Soto C. Cervera-Santiago J. L. (2021). Emotional intelligence measures: a systematic review. Health 9:1696. doi: 10.3390/healthcare9121696, 34946422 PMC8701889

[ref23] Budzanowska-Drzewiecka M. Tutko M. (2021). The impact of individual motivation on employee voluntary pro-environmental behaviours: the motivation towards the environment of polish employees. Manag. Environ. Qual. 32, 929–948. doi: 10.1108/MEQ-11-2020-0268

[ref24] Byrne B. M. (2013). Structural equation modeling with Mplus: Basic concepts, applications, and programming. New York: Routledge.

[ref25] Chang Y. C. Tsai Y. T. (2022). The effect of university students’ emotional intelligence, learning motivation and self-efficacy on their academic achievement—online English courses. Front. Psychol. 13:818929. doi: 10.3389/fpsyg.2022.818929, 35250754 PMC8888520

[ref26] Cheung T. Graham L. T. Schiavon S. (2022). Impacts of life satisfaction, job satisfaction and the big five personality traits on satisfaction with the indoor environment. Build. Environ. 212:108783. doi: 10.1016/j.buildenv.2022.108783

[ref27] Chiang J. (2025). A conceptual eighteen crucial factors development of self-determination theory in practice of online learning environment. Educ. Inf. Technol. 30, 10023–10038. doi: 10.1007/s10639-024-13227-4

[ref28] Chida Y. Steptoe A. (2008). Positive psychological well-being and mortality: a quantitative review of prospective observational studies. Psychosom. Med. 70, 741–756. doi: 10.1097/PSY.0b013e31818105ba, 18725425

[ref29] Çivilidağ A. Şekercioğlu G. (2017). Çok boyutlu iş motivasyonu ölçeğinin Türk kültürüne uyarlanması. Akdeniz İnsani Bilimler Dergisi 7, 143–156. doi: 10.13114/MJH.2017.326

[ref30] Conde-Pipó J. Melguizo-Ibáñez E. Mariscal-Arcas M. Zurita-Ortega F. Ubago-Jiménez J. L. Ramírez-Granizo I. . (2021). Physical self-concept changes in adults and older adults: influence of emotional intelligence, intrinsic motivation and sports habits. Int. J. Environ. Res. Public Health 18:1711. doi: 10.3390/ijerph18041711 33578889 PMC7916707

[ref31] Dağlı A. Baysal N. (2016). Yaşam doyumu ölçeğinin Türkçe’ye uyarlanması: Geçerlik ve güvenirlik çalişmasi. Elektronik Sosyal Bilimler Dergisi 15, 1250–1262. doi: 10.17755/esosder.263229

[ref32] Deci E. L. Ryan R. M. (1985). Intrinsic motivation and self-determination in human behavior. New York: Plenum Press.

[ref33] Deci E. L. Vallerand R. J. Pelletier L. G. Ryan R. M. (1991). Motivation and education: the self-determination perspective. Educ. Psychol. 26, 325–346.

[ref34] del Mar Salinas-Jiménez M. Salinas-Jiménez J. Artés J. (2024). “Income, motivation, and life satisfaction” in Encyclopedia of quality of life and well-being research. ed. Michalos A. C. (Cham: Springer International Publishing), 3413–3416.

[ref35] den Van Broeck A. Vansteenkiste M. Witte H. Soenens B. Lens W. (2016). Capturing autonomy, competence, and relatedness at work: construction and initial validation of the work-related basic need satisfaction scale. J. Occup. Organ. Psychol. 83, 981–1002. doi: 10.1348/096317909X481382

[ref36] Deniz M. E. Özer E. Işık E. (2013). Duygusal zekâ özelliği ölçeği–kısa formu: geçerlik ve güvenirlik çalışması. Eğitim ve Bilim 38, 407–419. doi: 10.15390/ES.2013.1180

[ref37] Diener E. (2009). “Subjective well-being” in The science of well-being: The collected works of Ed Diener. ed. Diener E. (Greenville NC: Springer), 11–58.

[ref38] Diener E. Chan M. Y. (2011). Happy people live longer: subjective well-being contributes to health and longevity. Appl. Psychol. Health Well-Being 3, 1–43. doi: 10.1111/j.1758-0854.2010.01045.x

[ref39] Diener E. D. Emmons R. A. Larsen R. J. Griffin S. (1985). The satisfaction with life scale. J. Pers. Assess. 49, 71–75. 16367493 10.1207/s15327752jpa4901_13

[ref40] Diener E. Lucas R. E. Oishi S. (2002). “Subjective well-being: the science of happiness and life satisfaction” in Handbook of positive psychology. eds. Snyder C. R. Lopez S. J.. (New York: Oxford University Press), 63–73.

[ref41] Diener E. Seligman M. E. (2002). Very happy people. Psychol. Sci. 13, 81–84. doi: 10.1111/1467-9280.00415, 11894851

[ref43] Dumitru C. Anagnoste M. Radu B. M. (2025). Exploring the link between emotional intelligence and academic performance in middle school students: a case study. BRAIN. Broad Res. Artificial Intelligence Neurosci. 16, 499–512. doi: 10.70594/brain/16.4/30

[ref44] Easterlin R. A. (2003). Explaining happiness. Proc. Natl. Acad. Sci. USA 100, 11176–11183. doi: 10.1073/pnas.1633144100, 12958207 PMC196947

[ref9006] Eccles J. S., Wigfield A. (2002). Motivational beliefs, values, and goals. Annual Rev. Psychol. 53, 109–132. 11752481 10.1146/annurev.psych.53.100901.135153

[ref45] Elçiçek Z. Han B. Yıldız S. (2022). Can teachers' job satisfaction be ensured despite economic inadequacies? The impact of positive psychological capital. Eur. J. Educ. Sci. 9, 1–10. doi: 10.19044/ejes.v9no1a1

[ref46] Extremera N. Fernández-Berrocal P. (2005). Perceived emotional intelligence and life satisfaction: predictive and incremental validity using the trait meta-mood scale. Pers. Individ. Differ. 39, 937–948. doi: 10.1016/j.paid.2005.03.012

[ref47] Extremera N. Salguero J. M. Fernández-Berrocal P. (2011). Trait meta-mood and subjective happiness: a 7-week prospective study. J. Happiness Stud. 12, 509–517. doi: 10.1007/s10902-010-9213-7

[ref48] Feather N. T. (2021). Expectancy-value approaches: present status and future directions. Expectations Actions, 395–420.

[ref49] Feraco T. Resnati D. Fregonese D. Spoto A. Meneghetti C. (2023). An integrated model of school students’ academic achievement and life satisfaction. Linking soft skills, extracurricular activities, self-regulated learning, motivation, and emotions. Eur. J. Psychol. Educ. 38, 109–130. doi: 10.1007/s10212-022-00601-4, 40477547 PMC8795749

[ref50] Fong C. J. (2022). Academic motivation in a pandemic context: a conceptual review of prominent theories and an integrative model. Educ. Psychol. 42, 1204–1222. doi: 10.1080/01443410.2022.2026891

[ref51] Freed W. J. (2022). “Biology of motivation, dopamine, and brain circuits that mediate pleasure” in Motivation and desire: A new way to think about why we do everything and its basis in neuroscience (Cham: Springer International Publishing), 105–119.

[ref9004] Gagné M. Forest J. Gilbert M. H. Aubé C. Morin E. Malorni A. (2010). The motivation at work scale: Validation evidence in two languages. Education. Psychol. Measure. 70, 628–646. doi: 10.1177/0013164409355

[ref52] Gkintoni E. Dimakos I. Nikolaou G. (2025). Cognitive insights from emotional intelligence: a systematic review of EI models in educational achievement. Emerg. Sci. J. 8, 262–297. doi: 10.28991/ESJ-2024-SIED1-016

[ref53] Goleman D. (1995). Emotional intelligence. Bantam.

[ref54] Goleman D. (1998). Working with emotional intelligence. Bantam.

[ref55] Gómez-Leal R. Holzer A. A. Bradley C. Fernández-Berrocal P. Patti J. (2022). The relationship between emotional intelligence and leadership in school leaders: a systematic review. Camb. J. Educ. 52, 1–21. doi: 10.1080/0305764X.2021.1927987

[ref56] Han B. Tösten R. Elçiçek Z. (2022). Public leadership behaviors of school principals: does it affect teacher motivation and job satisfaction in Turkish culture? Int. J. Public Leadersh. 18, 209–228. doi: 10.1108/IJPL-03-2021-0026

[ref57] Helmi A. F. (2021). Meta-analysis of the correlation between emotional intelligence and life satisfaction. Anatol. J. Educ. 6, 63–74. doi: 10.29333/aje.2021.626a

[ref58] Huebner E. S. Suldo S. M. Gilman R. (2006). “Life satisfaction” in Children's needs III: Development, prevention, and intervention. eds. Bear G. G. Minke K. M. (Maryland, USA: National Association of School Psychologists), 357–368.

[ref59] Huy Q. N. Shipilov A. V. (2012). The key to acquisitive action: how the structure of problem formulation shapes acquisition decisions. Organ. Sci. 23, 1103–1114.

[ref60] Inglehart R. Klingemann H. D. (2000). “Genes, culture, democracy, and happiness” in Culture and subjective well-being. eds. Diener E. Suh E. M. (London: The MIT Press), 165–184.

[ref61] Jose B. Thomas A. (2024). Navigating the research landscape of emotional and social ıntelligence among young adults: a bibliometric perspective. Cureus 16, 1–12. doi: 10.7759/cureus.59130, 38803730 PMC11129524

[ref62] Joseph D. L. Newman D. A. (2010). Emotional intelligence: an integrative meta-analysis and cascading model. J. Appl. Psychol. 95, 54–78. doi: 10.1037/a0017286, 20085406

[ref63] Karataş K. Han B. Oral B. (2025). The role of academic self-efficacy in the relationship between motivation and future time perspective of teacher candidates. Organiz. Psychol. 15, 18–32. doi: 10.17323/2312-5942-2025-15-2-18-32

[ref64] Khodabakhsh S. (2022). Factors affecting life satisfaction of older adults in Asia: a systematic review. J. Happiness Stud. 23, 1289–1304. doi: 10.1007/s10902-021-00433-x

[ref65] Kline R. B. (2010). “Promise and pitfalls of structural equation modeling in gifted research” in Methodologies for conducting research on giftedness. eds. Thompson B. Subotnik R. F. (Washington, DC: American Psychological Association), 147–169.

[ref66] Latham G. P. (2023). “Motivate employee performance through goal setting” in Principles of organizational behavior: The handbook of evidence-based management. eds. Locke E. Pearce C. (New Jersey: Wiley Press).

[ref67] Locke E. A. Latham G. P. (1990). A theory of goal setting and task performance. New Jersey: Prentice-Hall.

[ref68] Lu G. Xie K. Liu Q. (2022). What influences student situational engagement in smart classrooms: perception of the learning environment and students' motivation. Br. J. Educ. Technol. 53, 1665–1687. doi: 10.1111/bjet.13204

[ref69] Lyubomirsky S. King L. Diener E. (2005). The benefits of frequent positive affect: does happiness lead to success? Psychol. Bull. 131, 803–855. doi: 10.1037/0033-2909.131.6.803, 16351326

[ref70] Lyubomirsky S. Layous K. (2013). How do simple positive activities increase well-being? Curr. Dir. Psychol. Sci. 22, 57–62. doi: 10.1177/0963721412469809

[ref71] Malvaso A. Kang W. (2022). The relationship between areas of life satisfaction, personality, and overall life satisfaction: an integrated account. Front. Psychol. 13:894610. doi: 10.3389/fpsyg.2022.894610 36211891 PMC9532945

[ref72] Mardanov I. (2021). “Intrinsic and extrinsic motivation, organizational context, employee contentment, job satisfaction, performance and intention to stay” in Evidence-based HRM: a global forum for empirical scholarship, vol. 9. ed. Lange T. (Leeds: Emerald Publishing Limited), 223–240.

[ref9002] Markus H. R. Kitayama S. (1991). Culture and the self: Implications for cognition, emotion, and motivation. Psychol. Rev. 98, 224–253.

[ref73] Matthews G. Zeidner M. Roberts R. D. (2002). Emotional intelligence: Science and myth. Cambridge: MIT Press.

[ref74] Mayer J. D. Salovey P. (1997). “What is emotional intelligence?” in Emotional development and emotional intelligence: Implications for educators. eds. Salovey P. Sluyter D. (New York: Basic Books), 3–31.

[ref75] Mayer J. D. Salovey P. Caruso D. R. (2000). “Models of emotional intelligence” in Handbook of intelligence. ed. Sternberg R. J.. 2nd ed. (Cambridge: Cambridge University Press), 396–420.

[ref76] Mayer J. D. Salovey P. Caruso D. R. (2002). Mayer-Salovey-Caruso emotional intelligence test (MSCEIT) user's manual. London: MHS Publishers.

[ref9005] Mayer J. D. Salovey P. Caruso D. R. (2008). Emotional intelligence: New ability or eclectic traits? American Psychol. 63, 503–517. doi: 10.1037/0003-066X.63.6.503 18793038

[ref77] Meyer S. Grob A. Gerber M. (2021). No fun, no gain: the stress-buffering effect of physical activity on life satisfaction depends on adolescents' intrinsic motivation. Psychol. Sport Exerc. 56:102004. doi: 10.1016/j.psychsport.2021.102004

[ref78] Mikolajczak M. Luminet O. Leroy C. Roy E. (2007). Psychometric properties of the trait emotional intelligence questionnaire: factor structure, reliability, construct, and incremental validity in a French-speaking population. J. Pers. Assess. 88, 338–353. doi: 10.1080/00223890701333431 17518555

[ref79] Mirzaei M. Hoseini Shavoun A. Ahmari Tehran H. (2025). Enhancing student motivation in higher education: evidence-based strategies for effective teaching. Med. Educ. Bull. 6, 1005–1014. doi: 10.22034/meb.2025.523614.1108

[ref80] Nelson D. B. Low G. R. (2011). Emotional intelligence. London: Pearson Education, Inc.

[ref81] Niemiec C. P. (2024). “Intrinsic and extrinsic values” in Encyclopedia of quality of life and well-being research. ed. Maggino F. (Cham: Springer International Publishing), 3651–3654.

[ref82] Nieto Carracedo A. Gómez-Iñiguez C. Tamayo L. A. Igartua Perosanz J. J. (2024). Emotional intelligence and academic achievement relationship: emotional well-being, motivation, and learning strategies as mediating factors. Psicol. Educ. 30, 67–74. doi: 10.5093/psed2024a7

[ref83] O'Boyle E. H. Humphrey R. H. Pollack J. M. Hawver T. H. Story P. A. (2011). The relation between emotional intelligence and job performance: a meta-analysis. J. Organ. Behav. 32, 788–818. doi: 10.1002/job.714

[ref84] Özen E. Karaca N. (2021). Investigating learner motivation in online education in terms of self-efficacy and self-regulation. J. Educ. Technol. Online Learn. 4, 745–758. doi: 10.31681/jetol.1016530

[ref85] Pavot W. Diener E. (1993). Review of the satisfaction with life scale. Psychol. Assess. 5, 164–172.

[ref86] Petrides K. V. (2009). “Psychometric properties of the trait emotional intelligence questionnaire (TEIQue)” in Assessing emotional intelligence: Theory, research, and applications. eds. Parker J. D. A. Saklofske D. H. Stough C.. (Boston, MA: Springer US), 85–101.

[ref87] Petrides K. V. Furnham A. (2001). Trait emotional intelligence: psychometric investigation with reference to established trait taxonomies. Eur. J. Psychol. Assess. 17, 148–157. doi: 10.1027//1015-5759.17.3.148

[ref88] Poveda-Brotons R. Izquierdo A. Perez-Soto N. Pozo-Rico T. Castejón J. L. Gilar-Corbi R. (2024). Building paths to success: a multilevel analysis of the effects of an emotional intelligence development program on the academic achievement of future teachers. Front. Psychol. 15:1377176. doi: 10.3389/fpsyg.2024.1377176 38524292 PMC10957637

[ref89] Qin Y. Liu J. Wu D. (2023). The impact of emotional intelligence on life satisfaction among Chinese nurses: a chain mediating model. Front. Psychol. 14:1125465. doi: 10.3389/fpsyg.2023.1125465 36874842 PMC9982156

[ref90] Rogowska A. M. Meres H. (2022). The mediating role of job satisfaction in the relationship between emotional intelligence and life satisfaction among teachers during the COVID-19 pandemic. European J. Investigation Health, Psychol. Educ. 12, 666–676. doi: 10.3390/ejihpe12070050, 35877450 PMC9323296

[ref91] Ryan R. M. (2023). The Oxford handbook of self-determination theory. Oxford: Oxford University Press.

[ref92] Ryan R. M. Deci E. L. (2000). Self-determination theory and the facilitation of intrinsic motivation, social development, and well-being. Am. Psychol. 55, 68–78. 11392867 10.1037//0003-066x.55.1.68

[ref93] Ryan R. M. Deci E. L. (2017). Self-determination theory: basic psychological needs in motivation, development, and wellness. UK: Guilford Publications.

[ref94] Ryan R. M. Deci E. L. (2020). Intrinsic and extrinsic motivation from a self-determination theory perspective: definitions, theory, practices, and future directions. Contemp. Educ. Psychol. 61:101860. doi: 10.1016/j.cedpsych.2020.101860

[ref95] Salovey P. Mayer J. D. (1990). Emotional intelligence. Imagin. Cogn. Pers. 9, 185–211. doi: 10.2190/DUGG-P24E-52WK-6CDG

[ref96] Schunk D. H. (2023). “Self-regulation of self-efficacy and attributions in academic settings” in Self-regulation of learning and performance. eds. Schunk D. H. Greene J. A. Schunk D. H.. (Oxfordshire: Routledge), 75–99.

[ref97] Schutte N. S. Malouff J. M. Simunek M. McKenley J. Hollander S. (2001). Characteristic emotional intelligence and emotional well-being. Cognit. Emot. 16, 769–785. doi: 10.1080/02699930143000482

[ref98] Schutte N. S. Malouff J. M. Thorsteinsson E. B. Bhullar N. Rooke S. E. (2007). A meta-analytic investigation of the relationship between emotional intelligence and health. Pers. Individ. Differ. 42, 921–933. doi: 10.1016/j.paid.2006.09.003

[ref99] Seligman M. E. Steen T. A. Park N. Peterson C. (2005). Positive psychology progress: empirical validation of interventions. Am. Psychol. 60, 410–421. doi: 10.1037/0003-066X.60.5.410, 16045394

[ref100] Sharma N. Pandey S. (2024). “Emotional Intelligence” in Machine and deep learning techniques for emotion detection. eds. Rai M. Pandey J. K. (Hershey: IGI Global).

[ref101] Skinner E. A. Kindermann T. A. Vollet J. W. Rickert N. P. (2022). Complex social ecologies and the development of academic motivation. Educ. Psychol. Rev. 34, 2129–2165. doi: 10.1007/s10648-022-09714-0

[ref102] Steel P. Schmidt J. Shultz J. (2008). Refining the relationship between personality and subjective well-being. Psychol. Bull. 134, 138–161. doi: 10.1037/0033-2909.134.1.138, 18193998

[ref103] Supramanian K. Shahruddin R. Sekar M. (2021). Emotional intelligence using ability model in context of nursing and its impact on end-stage renal disease patients: a narrative review. J. Current Res. Rev. 13, 151–158. doi: 10.31782/IJCRR.2021.131609

[ref104] Travis B. Greaves J. (2009). Emotional intelligence appraisal: measuring emotional intelligence. US: TalentSmart.

[ref105] Van Kleef G. A. De Dreu C. K. Manstead A. S. (2009). An interpersonal approach to emotion in social decision making: the emotions as social information model. Adv. Exp. Soc. Psychol. 42, 45–96. doi: 10.1016/S0065-2601(10)42002-X

[ref106] Veenhoven R. (2012). World database of happiness: Continuous register of scientific research on subjective appreciation of life. Rotterdam: Erasmus University.

[ref107] Vilca-Pareja V. Ruiz L. de Somocurcio A. Delgado-Morales R. Medina Zeballos L. (2022). Emotional intelligence, resilience, and self-esteem as predictors of satisfaction with life in university students. Int. J. Environ. Res. Public Health 19:16548. doi: 10.3390/ijerph192416548 36554428 PMC9778840

[ref108] Wang Y. Wang H. Wang S. Wind S. A. Gill C. (2024). A systematic review and meta-analysis of self-determination-theory-based interventions in the education context. Learn. Motiv. 87:102015. doi: 10.1016/j.lmot.2024.102015

[ref109] Wood A. M. Joseph S. Maltby J. (2008). Gratitude uniquely predicts satisfaction with life: incremental validity above the domains and facets of the five factor model. Pers. Individ. Differ. 45, 49–54. doi: 10.1016/j.paid.2008.02.019

[ref110] Xu J. Choi M. C. (2023). Can emotional intelligence increase the positive psychological capital and life satisfaction of Chinese university students? Behav. Sci. 13:614. doi: 10.3390/bs13070614 37504060 PMC10376301

[ref9003] Xu Y. Wang Y. (2023). Job stress and university faculty members’ life satisfaction: The mediating role of emotional burnout. Front. Psychol. 14:1111434. doi: 10.3389/fpsyg.2023.1111434 36818103 PMC9930908

[ref111] Yang J. (2021). Understanding and enhancing Chinese TEFL teachers’ motivation for continuing professional development through the lens of self-determination theory. Front. Psychol. 12:768320. doi: 10.3389/fpsyg.2021.768320, 34899516 PMC8657765

[ref112] Zheng S. Masuda T. Matsunaga M. Noguchi Y. Ohtsubo Y. Yamasue H. . (2021). Cultural differences in social support seeking: the mediating role of empathic concern. PLoS One 16:e0262001. doi: 10.1371/journal.pone.0262001, 34969056 PMC8718000

